# Deciphering the Action of Perfluorohexyloctane Eye Drops to Reduce Ocular Discomfort and Pain

**DOI:** 10.3389/fmed.2021.709712

**Published:** 2021-10-26

**Authors:** Miguel Delicado-Miralles, Enrique Velasco, Ariadna Díaz-Tahoces, Juana Gallar, M. Carmen Acosta, Adolfo Aracil-Marco

**Affiliations:** ^1^Cellular and Systems Neurobiology Unit, Instituto de Neurociencias, Universidad Miguel Hernández-Consejo Superior de Investigaciones Científicas, San Juan de Alicante, Spain; ^2^The European University of Brain and Technology-Neurotech^EU^, San Juan de Alicante, Spain; ^3^Instituto de Investigación Sanitaria y Biomédica de Alicante, San Juan de Alicante, Spain

**Keywords:** ocular discomfort, ocular pain, dry eye, perfluorohexyloctane, blinking, tearing, cold thermoreceptors, corneal surface temperature

## Abstract

Perfluorohexyloctane (F6H8) eyedrops have been recently introduced in Europe as a product to treat dry eye disease, based on its ability to reduce tear film instability in Meibomian gland dysfunction and evaporative dry eye disease, although its mechanism of action is still unknown. In the present pilot study, we evaluated the effects of the ocular instillation of a single drop of commercial F6H8 eyedrops in 20 healthy humans (9 women/11 men), measuring: (a) Corneal surface temperature (CST) from infrared video images; (b) tear volume using phenol red threads; (c) blinking frequency; and (d) ocular surface sensations (cold, dryness, pricking, foreign body, burning, itching, gritty, eye fatigue, watering eyes, and light-evoked discomfort sensations; scored using 10 cm Visual Analog Scales), before and 5–60 min after F6H8 or saline treatment. CST decreased and tearing and blinking frequency increased significantly after F6H8 but not after saline solution. When applied unilaterally, CST decreased only in the F6H8-treated eye. No sensations were evoked after F6H8 or saline. The corneal surface temperature reduction produced by topical F6H8 does not evoke conscious ocular sensations but is sufficient to increase the activity of corneal cold thermoreceptors, leading to an increased reflex lacrimation and blinking that may relieve dry eye condition thus reducing ocular discomfort and pain.

## Introduction

The ocular surface is a unique exposed mucosa that must endure environmental conditions while maintaining its function and integrity (1). Upon their activation by environmental physical and chemical changes acting on their peripheral nerve endings, trigeminal sensory neurons innervating the ocular surface trigger protective responses such as blinking and tearing (2). In particular, there is strong evidence that TRPM8-mediated activation of corneal cold-thermoreceptors constitutes the afferent signal to the CNS for the regulation of tearing and blinking, mechanisms that allow to maintain and distribute moistness of the eye surface (3, 4).

Dry Eye Disease (DED), a condition that affects over 10% of people worldwide (5), is characterized by a loss of the so-called homeostasis of the tear film, that is, the disruption of the equilibrium of the chemical composition and functions of the tear film due to one or more of the underlying causes of dry eye (6). Due to the multi-etiological origin of DED, no specific treatments are available nowadays, and there is scarce scientific evidence on their effectiveness in the management of the disease. Artificial tears are commonly used by most DED patients (7) although some of them contain preservatives that are known to produce side effects (8).

Perfluorohexyloctane (F6H8) is a semifluorinated alkane liquid that has been used initially in ophthalmology as a long-term vitreous substitute (9). This compound is physically, chemically and physiologically inert, slightly amphiphilic, colorless and laser stable with a density higher than water, and very low surface and interface tensions (10). In addition, as it is a non-aqueous liquid, microbial growth is not possible and therefore, it does not need any preservative (8).

F6H8 applied topically in DED patients reduced their dry-eye associated symptoms in two prospective observational studies (8, 11). As F6H8 increased tear film breakup time and lipid layer thickness in DED patients, it has been proposed that F6H8 could prevent the increased evaporation that causes DED by forming an occlusive layer and reducing shearing forces of the eyelid during blinking (8, 11). This idea is reinforced by the observation that in rabbits, F6H8 improves the quality grade of the tear film lipid layer measured by hand-held interferometry (12). Additionally, in mild to moderate DED patients F6H8 transiently increases tear film thickness 10 min after its application (13). A more recent study showed that topical treatment with F6H8 does not induce changes in corneal endothelium and significantly reduce corneal staining in DED patients, supporting its effectiveness and safety (14).

These results, together with the low surface tension of the compound, led to conclude that the very small drop of F6H8 (about 10 μL) spread uniformly over the ocular surface upon application, forming a protective layer over the tear film and preventing its evaporation. However, the precise mechanisms that would explain the effects of F6H8 in DED are far from being clarified and still need investigation.

In a previous report, we found that F6H8 produces corneal surface temperature changes in tear-deficient guinea pigs (15), suggesting that F6H8 may be more than an inert molecule, forming a non-water mixable thin layer over the tears and reducing tear evaporation. We hypothesized that, in addition to preventing evaporation, F6H8 may facilitate heat exchange between corneal tissue and the environment, thus reducing corneal temperature and activating TRPM8 cold-thermosensitive channels of cold thermoreceptor nerves. In turn, the increased activity of corneal cold nerves will lead to an increase in tearing and blinking rate. To test this hypothesis, we have studied the ocular sensations evoked in a group of volunteers by topical instillation of F6H8, in parallel with its effects on tear production, blinking frequency, and corneal surface temperature measured by infrared thermography. Additionally, we performed a simple experiment to investigate the temperature transmittance of F6H8 as a first approach to understand the mechanism of action of this molecule.

## Materials and Methods

### Subjects

Twenty 20 young healthy volunteers (9 women, 11 men; mean age 24.1 ± 4.4 years, range 19–34 years) participated in this pilot study. After signing an informed consent, volunteers were subjected to a brief anamnesis and filled out an ocular surface discomfort index (OSDI) questionnaire adapted to Spanish-speaking people (16). Individuals with previous eye disease, ocular surgery, OSDI ≥12, as well as daily contact lens users or subjects that were receiving either ocular or systemic drugs were excluded. Participants were instructed to not consume any anti-inflammatory or pain-killer drug in the 48 h previous to the experiment. All experimental procedures were carried out according to the Spanish legal regulations and the Helsinki Declaration, and followed the protocol UMH.INJGa.01.14 approved by the Ethics Committee of the Universidad Miguel Hernández de Elche.

### Experimental Protocols

**Figure 1:**
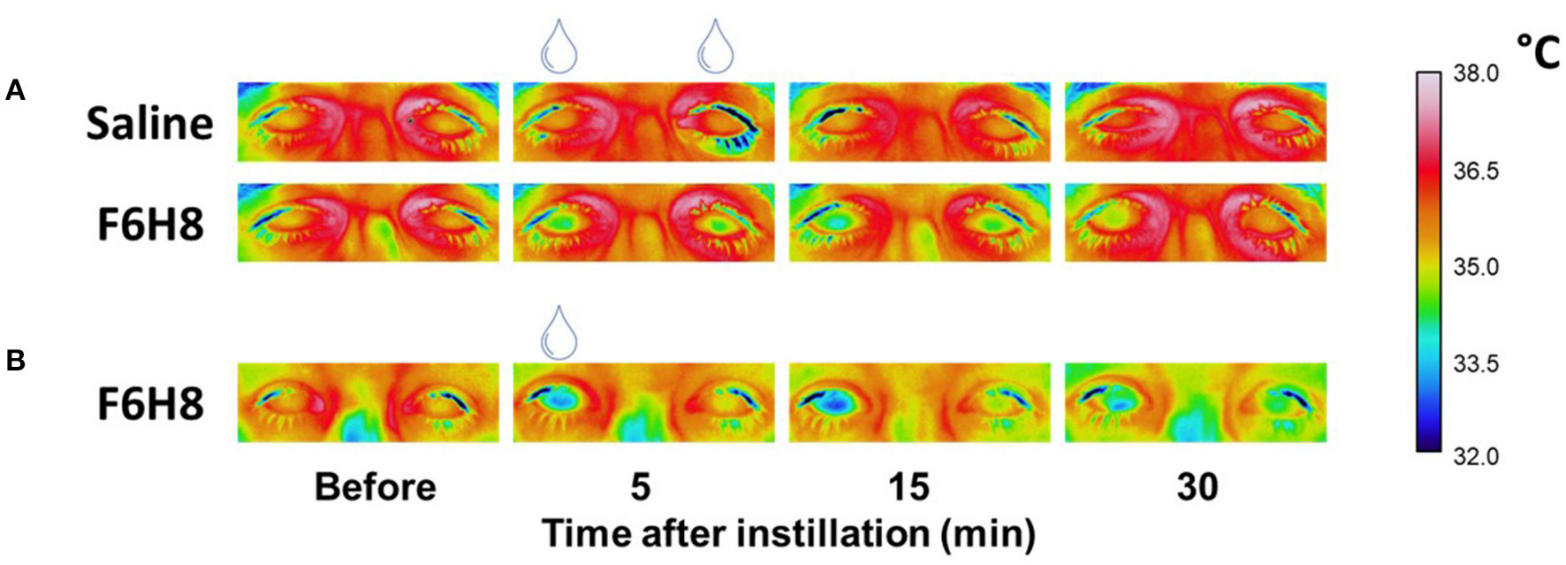
Representative examples of the infrared video images of both eyes taken to define the CST changes after bilateral topical application of saline or F6H8 **(A)** or unilateral (right eye) topical application of F6H8 **(B)**.

Two different experimental protocols were carried out (Figure 1) in the same room, under controlled temperature (24.2 ± 1.5°C, range 21.2–27.9°C) and partial humidity (42.4 ± 7.9%, range 22.6–55.0%). The position of the volunteers and experimenters, as well the distance from the face of the subject to the different objects in the room (video camera, air conditioning outlet, door, windows, etc.) was standardized to avoid any environmental variation along the procedures. As F6H8 does not induce corneal punctate (14), fluorescein corneal staining was considered not necessary. This way, the observed effects of F6H8 were not masked or affected by fluorescein or its excipients.

In experimental protocol 1 (Figure 1A), 13 participants received bilaterally a single 10 μL drop of F6H8 (EvoTears™, Brill Pharma S.L., Spain) or saline solution (NaCl 0.9 %, Braun Medical, S.A.) in two different sessions (application order at random). In experimental protocol 2 (Figure 1B), corneal surface temperature (CST) was measured in a separate group of 7 subjects (3 women and 4 men) before and after a single 10 μL drop of F6H8 instilled only on the right eye.

#### Protocol 1: F6H8 or Saline Solution Applied to Both Eyes

**Figure 2:**
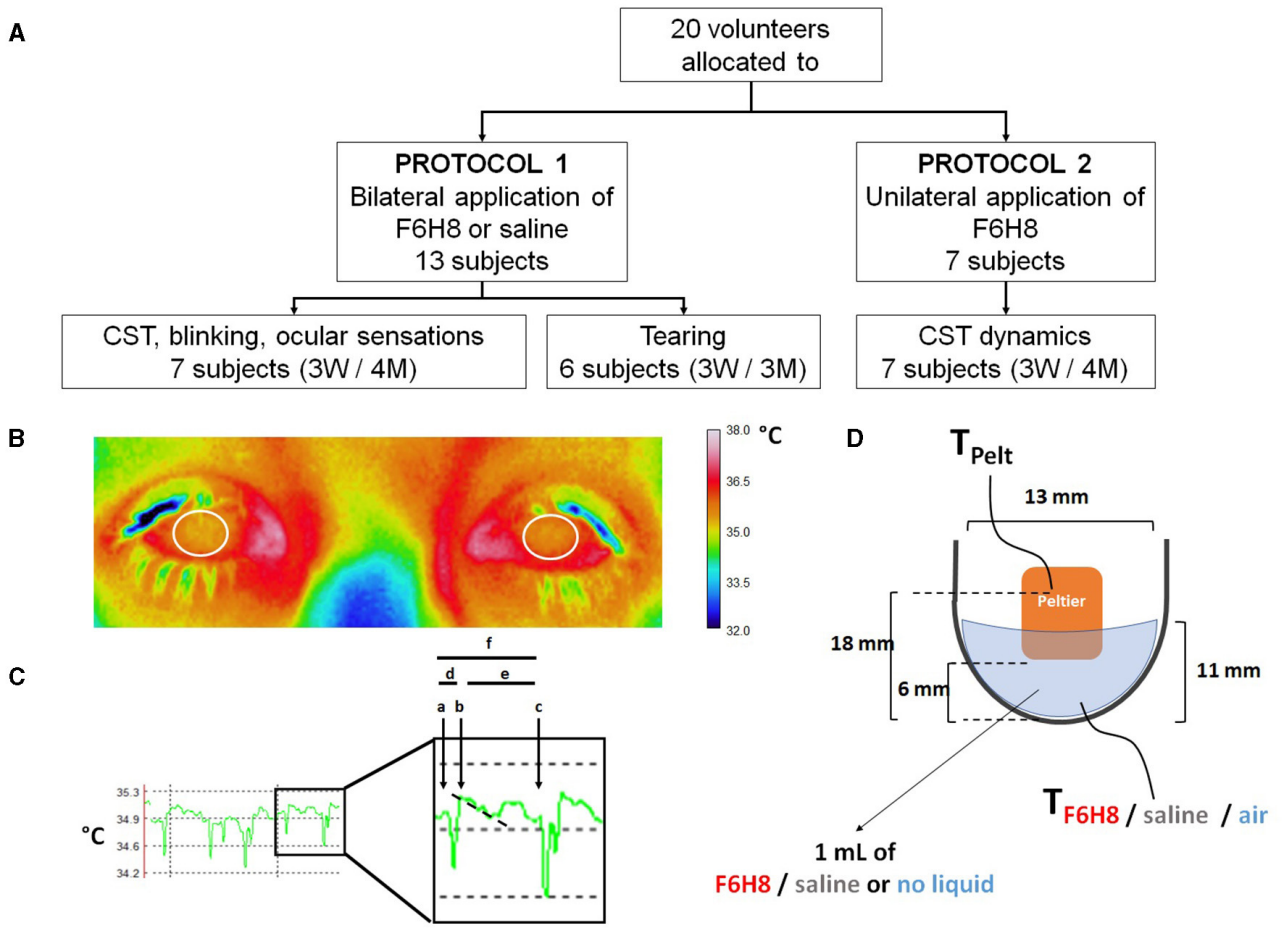
**(A)** Distribution of volunteer participants among the different experimental protocols of the pilot study. In experimental protocol 1, perfluorohexyloctane (F6H8) or saline solution were applied to both eyes, while in experimental protocol 2, F6H8 was applied only to the right eye. CST: corneal surface temperature. **(B)** CST was calculated from infrared thermographic video images. Temperature values of a 1 cm^2^ area of the corneal surface (white circumferences) were averaged to obtain the CST value at a defined time point. **(C)** Parameters measured from infrared thermographic video images of the corneal surface. Six interblink intervals were randomly selected and analyzed to obtain the following variables: (a) CST value immediately before a blink; (b) CST value immediately after blink; (c) CST value immediately before the next blink; (d) CST change during a blink, calculated as b-a; (e) CST change during the interblink interval, calculated as c-b; and (f) CST change between consecutive blinks, calculated as c-a. The time between a and b was considered as the duration of the blink movement. Also, the slope of the temperature decay during the first second of the IBI was calculated (dotted line). **(D)** Schematic representation of the experimental set-up used to measure the dynamics of temperature changes of F6H8 and saline solution during temperature changes induced with a Peltier cell placed inside the liquid in a tube. Temperature was measured with a thermocouple submerged into the liquid contained in a tube. TPelt: temperature of the Peltier cell measured with a PT100 sensor. TF6H8, TSaline and TAir: temperature measured with a thermocouple placed inside F6H8, saline solution, or an empty container, respectively.

Participants in protocol 1 were distributed in two different subsets (Figure 2A). In the first subset (protocol 1A) of 7 subjects (3 women and 4 men) CST, blinking frequency and ocular surface sensations were evaluated before and at different times (5, 15, 30, and 60 min) after F6H8 or saline bilateral treatment. In the second subset (protocol 1B) of 6 participants (3 women and 3 men), tearing was measured before and after F6H8 or saline.

#### Protocol 1A: CST, Blinking Rate and Ocular Sensations

**Measurement of corneal surface temperature**. Surface temperature of the ocular tissue was measured from video images taken with an infrared thermographic video camera (InfRec R300SR, Nippon Avionics Co. Ltd., Tokyo, Japan). The subject sat quietly with the head in a chin rest, fixing the gaze over the objective of the camera, placed at a fixed distance of 50 cm. Recording parameters (digital 1.6x zoom; 320 x 240 pixels; 60 frames per second; 0.96 emissivity) as well as data extraction (a circular area of 1 cm^2^ -range 0.93–1.01 cm^2^- specifically located over the cornea) were established using dedicated software (InfRec Analyzer NS9500 Standard, Nippon Avionics Co. Ltd.) (Figure 2B). Both eyes were simultaneously recorded for 1 min at different time points: before and 5, 15, 30, and 60 min after the corresponding topical treatment. At the beginning of the 1 min recording, subjects kept their eyes closed for 3 s and blink spontaneously afterwards. CST values calculated by averaging the temperature of 1 cm^2^ area of both corneas at the beginning of the last registered interblink period were considered the main parameter to define the effects of F6H8 and saline treatments on ocular surface temperature.

**Ocular surface sensations**. Immediately after the end of the 1-min IR video recordings performed before and at different times after F6H8 or saline treatment, subjects were asked to use separate 10 cm Visual Analog Scales (VASs; where 0 represents no sensation and 10 is the maximal sensation the subject can imagine) (17) to score the following sensations experienced at the ocular surface: cold, burning, dryness, pricking, foreign body sensation, itching, gritty, eye fatigue, watering eyes, and light-induced discomfort.

**Blinking frequency**. Immediately afterwards, the number of blinks was manually counted from direct observation of the subjects, who did not know that their blinks were being counted in order to avoid conditioning by the experimental situation (18). Volunteers were asked to read aloud the letters in a LogMar chart placed at 1 m distance, from left to right and from up to down. Blinking frequency (BF) was calculated as the number of blinks during the duration of the task for each subject. The average time needed to perform one complete reading of the chart was 24.6 ± 8.6 s, although depending on the subject it varied between 15 and 60 s. BF while performing this task was measured before, and at 5, 15, 30, and 60 min after the corresponding topical treatment.

#### Protocol 1B: Tear Volume

Tear volume was assessed before and 5, 15, and 30 min after the corresponding topical treatment using phenol red threads carefully placed during 30 s in the inferior conjunctival sac, near the temporal canthus. Tear volume was expressed as the length of wet thread, measured in mm using a rule.

#### Protocol 2: F6H8 or Saline Solution Applied Only to the Right Eye

CST was measured in both eyes before and at different time points after F6H8 instillation onto the right eye only, using the infrared thermography analysis described before. To further define the effects of F6H8, the following parameters were analyzed from the IR video recordings performed before, 5 and 60 min after treatment, averaging the values obtained from 6 interblink periods (Figure 2C): (a) CST value immediately before one blink; (b) CST value immediately after blink; (c) CST value immediately before next blink. From these values, (d) CST change during blink, (e) CST change during the interblink interval (IBI), and (f) CST change between consecutive blinks were calculated. Also, the slope of the temperature decay during the first second of the IBI was calculated. Additionally, the temperature of 1 cm^2^ of the eyelid skin was measured at the different time points before and after eyedrop treatment.

### Adaptation of F6H8 and Saline Solution to Temperature Changes

An ultrafine flexible temperature thermocouple (IT-23, Physitemp Instruments LLC, Clifton, NJ, USA) was placed at the bottom of an Eppendorf tube filled with 1 ml of F6H8 or saline solution, or empty of liquid (*n* = 4 observations per condition). Temperature was continuously recorded with a digital thermometer (BAT-12 Microprobe Thermometer, Physitemp Instruments LLC) (Figure 2D). Increases and decreases of temperature inside the tube were produced by a home-made temperature controller device whose Peltier cell was placed inside the tube. This device allows changing temperature between 15° and 50°C although only the temperature range close to the normal ocular surface temperature values were explored. From a resting Peltier temperature (T_Peltier_) around 34°C, temperature was increased by 3°C in a single step at an approximate rate of 0.030°C·s^−1^. After 8 min at 37°C, a 3°C cooling step was induced with the Peltier at a similar cooling rate. T_Peltier_ and temperature of the solution (T_F6H8_, T_saline_, or T_Air_) were recorded simultaneously and stored in a computer using a micro1401 CED interface and Spike2 software (both from Cambridge Electronic Devices, Ltd., Milton, Cambridge, UK) for further off-line analysis. As in the case of human measurements, experiments were made at a room temperature of 23–24°C and a partial humidity around 40%.

### Data Analysis

Power analysis for paired comparison analysis (matched pairs) was performed using Gpower^*^3.1 (19), considering an effect size of 1.5 (Cohen's d), a power of 0.8 and an α-error of 0.05. The minimum number of observations was established in *n* = 6, so that the sample size of participants in each subset of the experimental protocol (*n* = 6 or 7) was enough to achieve statistical significance.

Statistical analyses were performed using IBM SPSS Statistics for Windows (Version 25.0). Descriptive analysis was performed to detect possible outliers. Data distribution was studied with the Kolmogorov-Smirnoff test. Variances were compared using the Levene's test for Equality of Variances, when necessary. Normally distributed variables were compared with the paired Student's *t*-test, ANOVA or Repeated measurements ANOVA. Non-normally distributed parameters were compared with the Wilcoxon's test. Categorical variables were compared with the χ^2^ test. Unless otherwise indicated, data are presented as mean ± standard deviation (median ± interquartile range if non-normal). Statistical differences were accepted for *p* < 0.05. Graphs were made with SigmaPlot software v11.0 (Systat Software Inc., San Jose, CA, USA).

## Results

### Effects of Topical F6H8 and Saline Solution Applied to Both Eyes

We first studied the effects of bilateral topical instillation of a 10 μL drop of F6H8 on corneal surface temperature (CST), blinking rate and ocular surface sensations measured at different time points after treatment in 7 seven healthy young volunteers. Results were also compared with those obtained after bilateral instillation of an aqueous solution (saline solution).

#### Ocular Surface Sensations

Sensations of cold, dryness, burning, pricking, foreign body sensation, itching, gritty, eye fatigue, watering eyes, and light-induced discomfort experienced by the volunteers were evaluated before and at different times after the corresponding treatment. Overall, no conscious ocular sensations were evoked by F6H8 or saline treatment at any of the studied time points, being 0 the median of the scored values. As an example, Table 1 shows the VAS values of the ocular sensations scored 5 min after bilateral topical treatment with F6H8 or saline. In addition, no differences in the proportion of subjects reporting any sensation were found between F6H8 and saline treatments (Table 1), although 5 out of the 7 subjects were able to differentiate F6H8 from saline. Two subjects also reported blurry vision for a few seconds after F6H8 application.

**Table 1:** Ocular surface sensations reported 5 min after bilateral topical treatment with a 10 μL drop of F6H8 or saline solution.

	**F6H8**		**Saline**	
**Sensations**	**Sensation intensity**	**Responding subjects**	**Sensation intensity**	**Responding subjects**
Cold	0 (0.0)	1/7	0 (0.0)	1/7
Dryness	0 (0.0)	0/7	0 (0.0)	1/7
Burning	0 (0.0)	1/7	0 (3.2)	2/7
Pricking	0 (0.0)	0/7	0 (0.0)	0/7
Foreign body	0 (6.0)	2/7	0 (7.1)	1/7
Itching	0 (0.0)	1/7	0 (6.8)	3/7
Gritty	0 (4.8)	1/7	0 (0.0)	1/7
Eye fatigue	0 (0.0)	1/7	0 (0.0)	0/7
Tearing	0 (0.0)	0/7	0 (8.4)	2/7
Light-evoked discomfort	0 (0.0)	0/7	0 (1.3)	1/7

*Data shown on each column are: median (IQR) of VAS units; number of responding/total number of explored subjects. No significant differences were found between F6H8 and saline-treated groups for any sensation (Wilcoxon Signed Rank test and chi square test)*.

#### Corneal Surface Temperature

**Figure 3:**
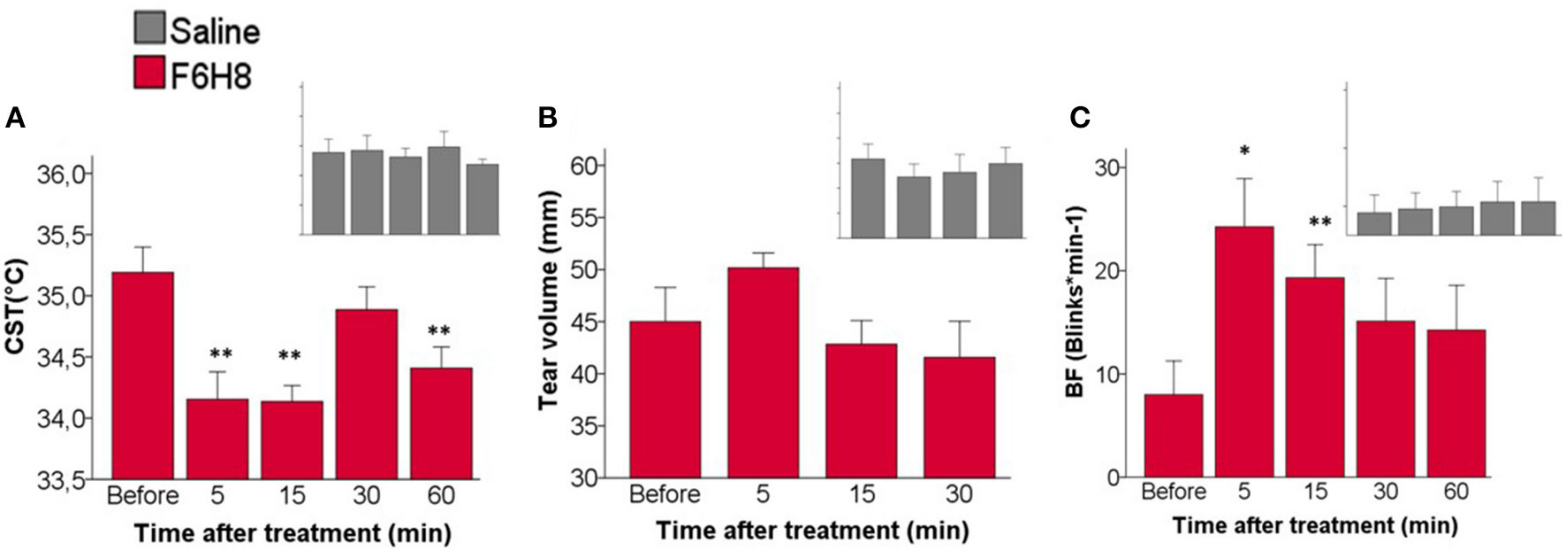
Effects of topical instillation of F6H8 (red) or saline solution (insets in gray) on both eyes. **(A)** CST at the beginning of the last interblink interval measured from infrared thermographic images, *p* = 0.001, Repeated Measurements ANOVA; **p < 0.01 Dunnett's test *n* = 7. **(B)** Tear volume measured with phenol red threads, no significant differences, Repeated Measurements ANOVA, *n* = 6. **(C)** Blinking frequency (BF), *p* < 0.005, Repeated Measurements ANOVA; *p < 0.05, **p < 0.01, Dunnett's test, *n* = 7.

At different times after bilateral topical treatment (Figure 1A), CST values were calculated by averaging the temperature of 1 cm^2^ of corneal surface (Figure 2B) in infrared thermographic images taken immediately after eye opening (Figure 2C, parameter b, see methods). CST was significantly decreased after F6H8 (*p* = 0.001, Repeated Measurements ANOVA; *p* = 0.001, 0.008, 0012 at 5, 15 and 60 min, respectively, in comparison with the value before treatment, *post hoc* Dunnett's test; *n* = 7) (Figure 3A). In contrast, bilateral instillation of saline solution did not modify CST at any of the different time point after treatment (Figures 1A, 3A, inset).

#### Tear Volume

No significant changes in the volume of tears collected with phenol red threads were found after saline treatment (*p* = 0.640, Repeated measures ANOVA; *n* = 6) (Figure 3B). Tear volume was slightly increased only at 5 min after F6H8, although the change was not statistically significant (*p* = 0.151, Repeated Measurements ANOVA; *n* = 6).

#### Blinking Frequency

Bilateral application of saline did not affect blink frequency at any studied time point (Figure 3C inset). In contrast F6H8 significantly increased BF (*p* = 0.004, Repeated Measurements ANOVA; *p* = 0.015 and 0.008 at 5 and 15 min, respectively, in comparison with the value before treatment, *post hoc* Dunnett's test; *n* = 7) (Figure 3C).

### Effects of Unilateral Administration of F6H8 on CST

This set of experiments was performed to further describe the cooling effect of F6H8. In a separate group of volunteers (*n* = 7, see Methods), CST was measured before and after a single drop of F6H8 applied only onto the right eye in order to compare the dynamics of the temperature change during the interblink intervals (IBIs) in the treated eye, in comparison with the untreated, fellow eye. For that purpose, the evolution of CST values along the interblink interval was analyzed (See Methods and Figure 2C for details).

**Figure 4:**
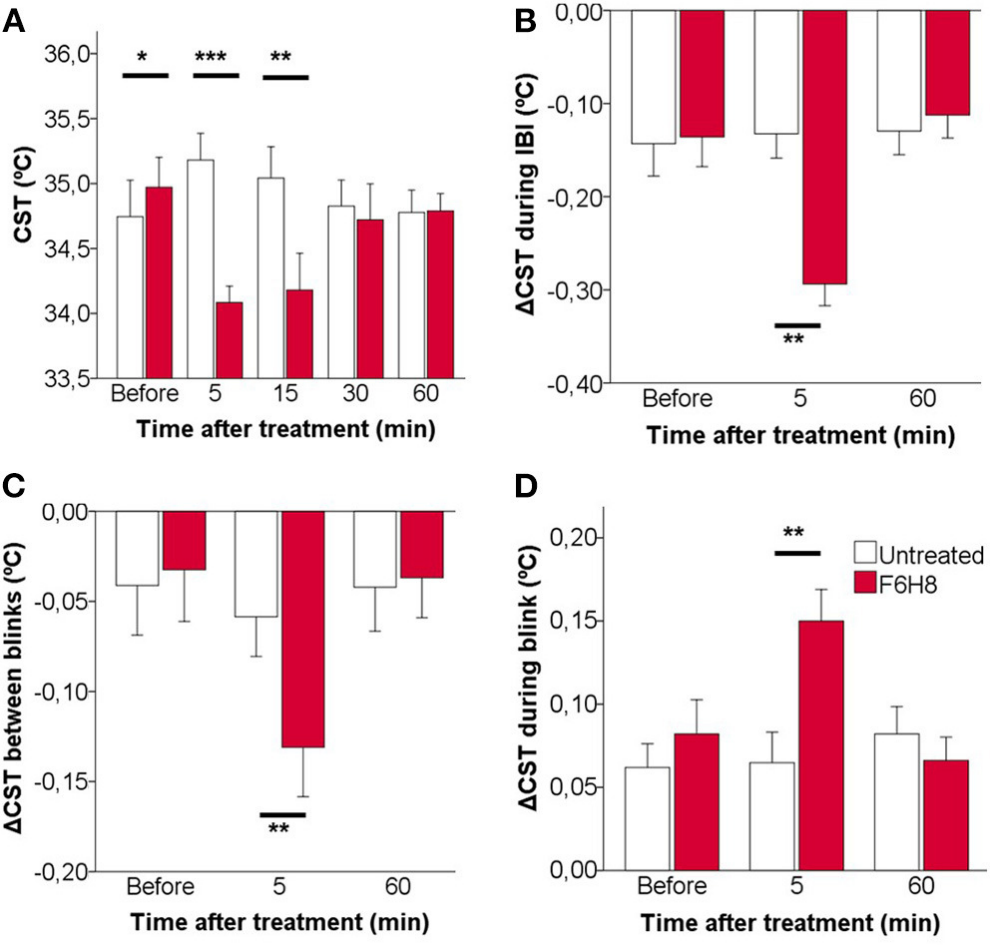
Effects of topical instillation of F6H8 only on the right eye in comparison to the untreated left eye, on CST values measured from infrared thermographic images. **(A)** CST measured immediately after blink, that is, at the beginning of the interblink interval. **(B)** CST change during the IBI. **(C)** CST change between consecutive blinks. **(D)** CST change during blink. Data are mean ± standard deviation, *n* = 7; *p < 0.05, **p < 0.01, ***p < 0.001, paired *t*-test.

As expected, CST values at the beginning of the IBI were reduced after F6H8 (*p* = 0.001, repeated measures ANOVA; *p* = 0.002 and 0.007 for the values obtained at 5 and 15 min after treatment, respectively, compared with pre-treatment values, *post hoc* Dunnett's test; *n* = 7) with a maximal effect 5 min after treatment (Figure 4A). On the contrary, in untreated eyes, no significant changes of the CST values at the beginning of the IBI were found at any explored time point (Figure 4A). The CST values obtained at the beginning of the IBI were significantly lower in eyes receiving F6H8 than in untreated eyes at 5 and 15 min after treatment (*p* = 0.001 and 0.023, respectively, paired *t*-test; *n* = 7) (Figure 4A). To define if the cooling effect was restricted to the eye surface, we also measured the temperature of the eyelid skin, finding that it was not significantly modified neither in the F6H8-treated (35.01 ± 0.61°C vs. 35.3 ± 0.44°C, before and 5 min after, respectively; *p* = 0.072, paired *t*-test) or untreated eye (34.98 ± 0.57°C vs. 35.37 ± 0.53°C; *p* = 0.052).

To describe in more detail the effects of F6H8 on ocular surface temperature, the dynamics of CST change along an IBI was studied from the infrared video images taken before and 5 min after administration (when the maximal temperature reduction was obtained), as well as 60 min after F6H8 treatment (Figures 4B–D). In addition to starting from a lower temperature at the beginning of the IBI (Figure 4A), the decay of temperature during IBI was more prominent in the F6H8-treated eyes at 5 min after treatment, and recovered basal values afterwards (Figure 4B, red bars). This effect was not present in the untreated eye (Figure 4B, empty bars). During the IBI, eyes treated with F6H8 cooled faster than untreated eyes, as reflected by the faster slope of the temperature decay during the first second (−0.078 ± 0.16°C/s and −0.165 ± 0.82°C/s, before and 5 min after F6H8, respectively; *p* < 0.01, paired *t*-test). On the contrary, the slope of temperature decay during IBI did not change significantly in the untreated eye (−0.061 ± 0.145°C/s and −0.082 ± 0.101°C/s, before and 5 min, respectively; *p* = 0.437).

The reduction of CST induced by F6H8 was present immediately after its application, although the magnitude of the cooling effect was increasing with time during the first 15 min after treatment (Figures 1B, 4A). The increasing cooling during this time was evidenced by the significant differences obtained when comparing CST values of consecutive blinks (Figure 4C).

We then compared CST values obtained immediately before and after a blink to measure the magnitude of warming of the ocular surface that occurred during the time when the eyes were closed. This CST increase produced during blink was significantly larger in the eyes receiving F6H8 than in the contralateral, untreated eyes (Figure 4D). As this warming of the ocular surface is produced by heat transference between the vascularized palpebral conjunctiva and the avascular corneal tissue, we speculate whether the increased warming during blink was due to a longer duration of the eye closure in F6H8-treated eyes. We then used the IR video recordings to measure blink duration, finding that the duration of eye closure during blink was not modified after F6H8 (0.59 ± 0.14 ms vs. 0.65 ± 0.18 ms, before and 5 min after F6H8, respectively; *p* = 0.128, paired *t*-test).

### Thermal Adaptation of F6H8 and Saline Solution to Temperature Changes

**Figure 5:**
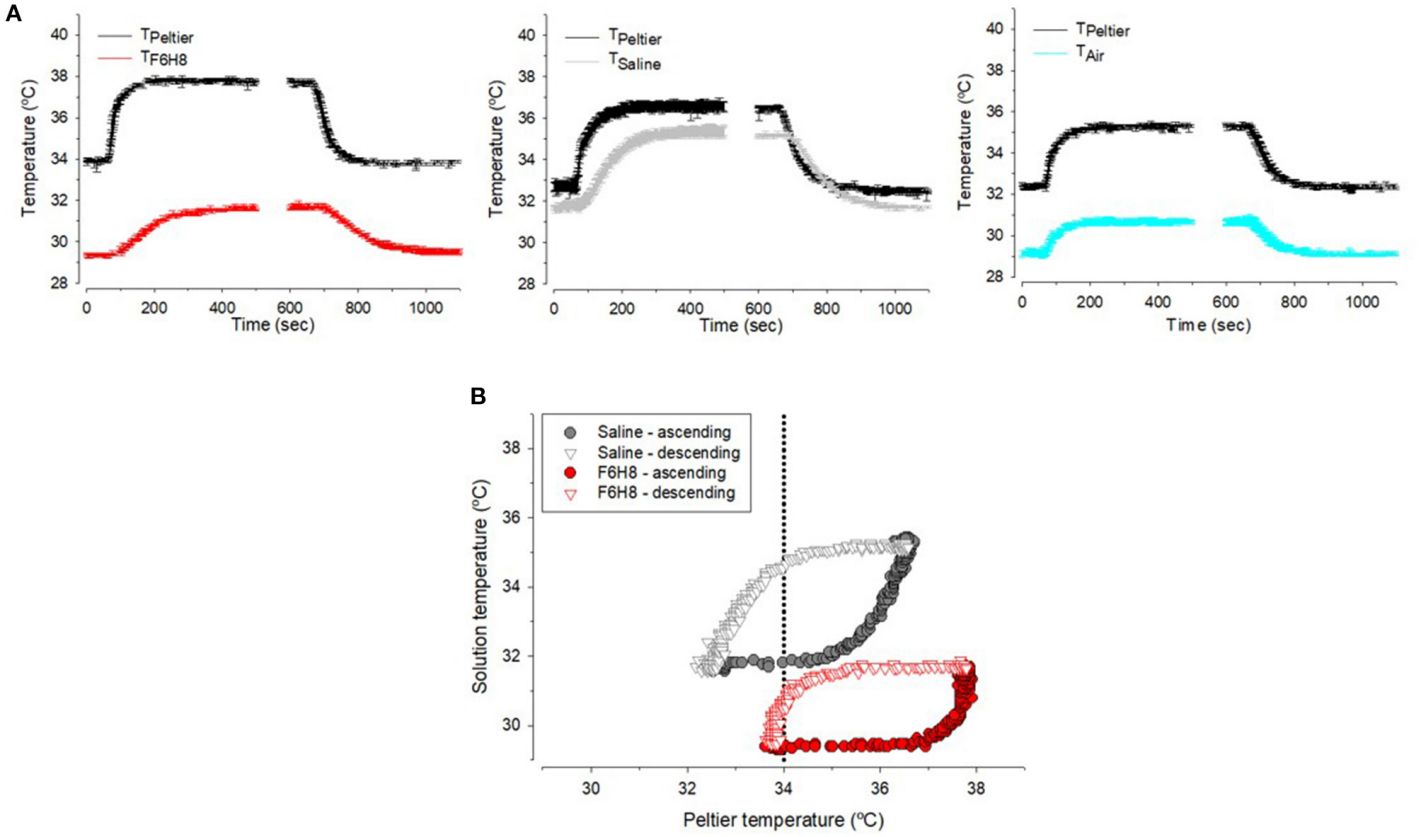
**(A)** Sample recordings of the temperature measured inside the tube filled with F6H8, saline or empty (air) during experimental warming or cooling in the conditions explained in method and Figure 1D. **(B)** Hysteresis curves during warming (closed symbols) or cooling (open symbols) inside saline (gray) and F6H8 (red).

Measurements done with a thermoprobe using the experimental setup described in Figure 2D showed that for a sustained Peltier cell temperature (T_Peltier_) around 33–34°C and a room temperature around 23°C, temperature of the saline solution (T_saline_) placed in a tube was close to 32°C, that is, around 1°C lower than T_Peltier_ (Figure 5A). Temperature of F6H8 (T_F6H8_) in this condition was around 29°C, that is, around 4–5°C lower than T_Peltier_ (Figure 5A). In this regard, T_F6H8_ behaved similarly to the temperature inside the tube measured without any liquid (T_air_), about 4°C lower than T_Peltier_ (Figure 5A). When changing the Peltier temperature, the profiles of T_saline_, T_F6H8_, and T_air_ followed the changes of T_Peltier_ (Figure 5A), although maintaining the difference described above. Comparison of T_saline_ and T_F6H8_ during ascending and descending temperature steps showed two different hysteresis patterns (Figure 5B). T_saline_ exhibited a slower rate of either increasing or decreasing temperature in comparison with T_F6H8_. Furthermore, the warming rate and cooling rate of each substance showed that F6H8 tended to cool down faster than saline. Moreover, under our experimental conditions, T_F6H8_ cooling rate was higher around 34°C (Figure 5B).

## Discussion

During the last 5 years, perfluorohexyloctane has been used as an alternative treatment of DED, particularly for its evaporative form due to Meibomian gland dysfunction (MGD) (8, 11, 13, 20). After 4–8 weeks of treatment with F6H8, MGD patients show an increase of the tear film and the lipid layer thickness (13), and Schirmer I test and Tear Film Break-Up Time (TFBUT) values, as well as a reduction of OSDI scores (8, 11). These data supported the idea that F6H8 is effective to treat evaporative forms of DED by improving the lipid layer of the tear film, and subsequently reducing tear evaporation and increasing tear film stability. This is a conceivable mechanism of action, because dry eye patients show an increased tear evaporation rate (21) and, due to its low surface tension, the F6H8 liquid state can act as a surfactant, forming monolayers at the water/air interphase (22, 23). F6H8 incorporation into artificial lipid systems mimicking the lipid layer of the tear film does not affect the tear film interface properties and restores the fluidity of these artificial lipid layers (24). Moreover, when applied onto healthy rabbit corneas, F6H8 spreads over larger areas than saline with lower viscosity (12). Therefore, F6H8 may contribute to restore, at least partially, the altered tear film lipid layer in evaporative DED patient.

To the best of our knowledge our observations are the first demonstration that, in addition, topical application of F6H8 onto the human eye decreases for several minutes the corneal surface temperature. Five min after administration of F6H8, CST decreased about −0.7°C in all the studied subjects. This ocular surface cooling occurs in parallel to an increase in tearing and blinking frequency that cannot be associated to the activation of the nociceptive corneal nerve fibers responsible to reflex blinking and tearing (25, 26) because any conscious sensation was evoked by F6H8.

The biophysical mechanism explaining the cooling effect of F6H8 is unknown. Cooling of the ocular surface after eye opening has been related to tear evaporation rate. Thus, the possibility exists that the compound would increase it. However, to the best of our knowledge tear evaporation after topical F6H8 administration has not been measured neither in evaporative DED patients, nor in healthy eyes. Only in an experimental model in healthy rabbit eyes *in vivo*, Agarwal et al. have described an acute biphasic effect of F6H8 in the percentage of change of tear evaporation from baseline (12). They noticed that although tear evaporation rate slightly and transiently increased by 5 min after F6H8 instillation, it tended to decrease 60–90 min afterwards, reaching values even below baseline. As the increase of tear evaporation occurred also after application of saline, these authors attributed this finding to the increased tear volume and, possibly, to the transient alteration of the tear film structure due to the instillation itself. An alternative mechanism could be the evaporation of the molecule itself, although F6H8 exhibits a low evaporation rate compared with other semifluorinated alkane molecules. When tested *in vitro*, <1.5% evaporated within 1 h, and more than 50% of the initial volume remained unevaporated after 24 h, both at 35°C (27). However, we cannot exclude that an increased fluid evaporation rate would explain, at least in part, the cooling effect found after F6H8 treatment.

According to Fourier's law of heat conduction, heat flow is inversely proportional to the thickness of the material and directly proportional to (a) the heat diffusion area; (b) the temperature gradient; and (c) the specific thermal conductivity constant of materials. In our experiments we assumed that the temperature gradient among the inner parts of the eye globe, the exposed area of the corneal tissue and the room environment keep constant, and that if F6H8 would cause an increase of the tear film thickness, this would represent a negligible increase of the total distance among the inner parts of the eye and the environmental air. Therefore, it can be hypothesized that a mechanism for the cooling effect of F6H8 would be an increase of the tear film thermal conductivity after the incorporation of F6H8 to the outermost tear film layer. In the presence of F6H8, we observed an increase of the ocular surface warming produced with blink (during the time that the eye is closed) and a faster decay of CST during the IBI. Interestingly, when measuring the thermal adaptation of F6H8 to temperature changes in a quite naïve experiment, we observed that the temperature measured inside liquid F6H8 tends to be between the temperature imposed by the Peltier cell and that of the environmental room air. Despite the absence of experimental data on the heat transmissivity properties of the molecule, it can be speculated that an increase of thermal conductivity and the subsequent increase of heat loss from the ocular surface to the environment is produced after topical administration of F6H8. However, an effect on radiative cooling cannot be ruled out with the present set of experiments.

In the present experiments we have not studied the time course of F6H8 removal from the ocular surface. Despite that, it seems reasonable that, at least partially, the compound could be drained continuously together with tear fluid, thus explaining the attenuation of the cooling effect of F6H8 with time. Previous studies estimated that the basal turnover of the tear film lipid layer occurs at an approximated rate of 1% per min in healthy humans (28). Therefore, if F6H8 is homogeneously distributed along the lipid layer and both are drained together, 30 min after F6H8 application it would be expected that 30% of the compound have been removed from the front of the eye. This value could be even higher, given that the molecule has a specific-gravity greater than water (9, 29). Thus, when the head is in vertical position -as in our experiments- F6H8 would tend to be accumulated in the lower part of tear film and the inferior tear meniscus, which could accelerate its draining. Despite deeper studies on the spatiotemporal dynamics of F6H8 and its distribution and removal from the ocular surface would be welcomed, existing literature on the precorneal residence of a F6H8 drop in an *ex vivo* model of porcine eye shows a rapid drop of 36% of the substance in the precorneal space during the first 10 min after its application. Interestingly, from that moment on, F6H8 elimination slowed down and 56% is still in the precorneal space 1 h after application (27), and even tends to accumulate in the corneal epithelium (27, 30).

The F6H8-induced cooling of the ocular surface was produced in parallel with transient increases in tearing and blinking. Considering the role of corneal cold-thermoreceptors on basal tear production and blinking (3, 4), it seems conceivable that the changes in the activity of this population of trigeminal sensory neurons would be signaling the F6H8-induced CST reduction, thus inducing reflex changes in tearing and blinking. Since the classical observations by Mapstone (31), both blinking and tearing are considered as physiologic reflex responses that counteract the ocular surface cooling produced during eye opening. In our experiments we found prominent and long-lasting effects of F6H8 in blinking frequency. We also confirmed that along a blinking cycle, the eye is closed about 6% of the time and open about 94% of the time, a long period when the cornea is losing heat to the environment. Blinking may counteract CST cooling by three mechanisms: (a) passive prevention of heat loss; (b) heat transfer from the eyelids to the ocular surface during blink; and, (c) re-layering of the warm tear film over the cornea (31). F6H8 increased blinking rate and reduced IBI duration therefore reducing the time that the ocular surface is losing heat to the environment due to the cornea-air temperature gradient. In the same direction, a slight increase of the eye-closure time was observed, therefore favoring the heat exchange from the lids to the cornea. However, both processes were not enough to counteract the net cooling effect that is possibly related to the spreading of the compound over the whole tear film with each blink and the subsequent F6H8 evaporation. Increased blink and tearing rate are due to an increase in the TRPM8-dependent activity of cold thermoreceptor neurons, whose central axon projections have synapses with second order neurons of the trigeminal brainstem complex (32). The corneal nerve endings of these thermosensitive cold neurons are activated by cooling and tear hyperosmolarity (33), and also by the continuous oscillatory changes of temperature and wetness produced in the front of the eyes while they are open. Psychophysical experiments showed that a corneal cooling between 1 and 2°C is needed to evoke conscious sensations of cooling, while reducing the corneal temperature beyond these values elicits sensations of irritation (17, 25). The increased neural activity evoked in cold thermoreceptor neurons, especially in those belonging to the high background-low threshold subtype (HB-LT), by the small temperature and/or osmolarity changes produced during the interblink interval (expected to be around 0.5°C) is sufficient to evoke blink reflex (34), while more intense corneal cooling recruit the low background-high threshold (LB-HT) cold thermoreceptor endings, whose activation, together with that of nociceptive nerve endings, is claimed to evoke irritation and pain sensations (35). In the present experiments we found that F6H8 reduces the corneal temperature ~1°C and also induces a fast and intense further cooling of the corneal surface during the interblink interval, two times larger in F6H8-treated eyes than in untreated or saline-treated eyes. We propose that this corneal temperature drop produced by F6H8 increases the firing of HB-LT cold thermoreceptor nerve endings to a level enough to reflexively increase blinking rate and tear production, although not enough to evoke cooling sensations arising from the ocular surface. The absence of conscious sensations after F6H8 may be also explained by the higher tear film stability produced by the molecule (8, 11). The F6H8 layer formed over the tear film would reduce aqueous tear evaporation and prevents the local production of tear hyperosmolarity and drying spots that are leading to the activation of corneal nerve endings and development of ocular sensations of dryness and irritation (36).

In summary, we described here the unknown long-lasting cooling effect of F6H8 when applied topically onto the healthy ocular surface. This effect was paralleled by thermal homeostatic responses to protect the avascular ocular surface, such as the increase of tearing and blinking, both reflex responses driven by the TRPM8-mediated activation of corneal cold-thermoreceptors in response to ocular surface cooling. Besides this temperature reduction, F6H8 increases tear film stability and thickness, which limits the production of the local osmolarity changes underlying the genesis of ocular sensations. As a concluding remark, F6H8 instilled onto the eye reduces corneal surface temperature enough to increase tearing and blinking rate but not to evoke conscious sensations of ocular discomfort. The increased tear volume more frequently redistributed over the ocular surface helps to prevent corneal dryness and contributes to the clinical benefits of F6H8 in DED and other ocular surface pathologies.

## Data Availability Statement

The original contributions presented in the study are included in the article/supplementary material, further inquiries can be directed to the corresponding author/s.

## Ethics Statement

The studies involving human participants were reviewed and approved by Órgano Evaluador de Proyectos de la Universidad Miguel Hernández de Elche. The patients/participants provided their written informed consent to participate in this study.

## Author Contributions

MD-M and EV acquired and interpreted the data. EV and JG conceived and designed the work. AD-T and AA-M contributed to design the experiments. MCA and AA-M equally contributed to supervise the work. All authors contributed to the article and approved the submitted version.

## Funding

This work was funded by the Spanish Agencia Estatal de Investigación and the European Regional Development Fund grants SAF2017-83674-C2-1-R and SAF2017-83674-C2-2-R, the Generalitat Valenciana Excellence Program grant PROMETEO/2018/114, Predoctoral fellowships ACIF/2019/054 from GV (MD-M) and FPU16/00283 from Spanish Ministry of Universities (EV), and PID2020-115934RB-I00/AEI/10.13039/501100011033.

## Conflict of Interest

The authors declare that the research was conducted in the absence of any commercial or financial relationships that could be construed as a potential conflict of interest.

## Publisher's Note

All claims expressed in this article are solely those of the authors and do not necessarily represent those of their affiliated organizations, or those of the publisher, the editors and the reviewers. Any product that may be evaluated in this article, or claim that may be made by its manufacturer, is not guaranteed or endorsed by the publisher.
